# A Machine Vision-Based Method Optimized for Restoring Broiler Chicken Images Occluded by Feeding and Drinking Equipment

**DOI:** 10.3390/ani11010123

**Published:** 2021-01-08

**Authors:** Yangyang Guo, Samuel E. Aggrey, Adelumola Oladeinde, Jasmine Johnson, Gregory Zock, Lilong Chai

**Affiliations:** 1 Department of Poultry Science, University of Georgia, Athens, GA 30602, USA; yangyang.guo@uga.edu (Y.G.); saggrey@uga.edu (S.E.A.); ade.oladeinde@usda.gov (A.O.); jcj36120@uga.edu (J.J.); gregzock@uga.edu (G.Z.); 2 U.S. National Poultry Research Center, USDA-ARS, Athens, GA 30605, USA

**Keywords:** broiler chicken, machine vision, image restoring, precision poultry farming

## Abstract

**Simple Summary:**

The equipment in the poultry house can occlude top view images of broiler chickens and limit the efficiency of vision-based target detection. In this study, we sought to improve the efficiency of a previously developed method to detect and restore broiler chicken areas blocked by feeders and drinkers. To do this, we developed and tested linear and elliptical fitting restoration methods under different occlusion scenarios to restore occluded broiler chicken areas. The restoration method correctly restored the occluded broiler chicken area >80% of the time. This study provides a practical approach to enhancing the image quality in applying a machine vision-based method for monitoring poultry health and welfare.

**Abstract:**

The presence equipment (e.g., water pipes, feed buckets, and other presence equipment, etc.) in the poultry house can occlude the areas of broiler chickens taken via top view. This can affect the analysis of chicken behaviors through a vision-based machine learning imaging method. In our previous study, we developed a machine vision-based method for monitoring the broiler chicken floor distribution, and here we processed and restored the areas of broiler chickens which were occluded by presence equipment. To verify the performance of the developed restoration method, a top-view video of broiler chickens was recorded in two research broiler houses (240 birds equally raised in 12 pens per house). First, a target detection algorithm was used to initially detect the target areas in each image, and then Hough transform and color features were used to remove the occlusion equipment in the detection result further. In poultry images, the broiler chicken occluded by equipment has either two areas (TA) or one area (OA). To reconstruct the occluded area of broiler chickens, the linear restoration method and the elliptical fitting restoration method were developed and tested. Three evaluation indices of the overlap rate (OR), false-positive rate (FPR), and false-negative rate (FNR) were used to evaluate the restoration method. From images collected on d2, d9, d16, and d23, about 100-sample images were selected for testing the proposed method. And then, around 80 high-quality broiler areas detected were further evaluated for occlusion restoration. According to the results, the average value of OR, FPR, and FNR for TA was 0.8150, 0.0032, and 0.1850, respectively. For OA, the average values of OR, FPR, and FNR were 0.8788, 0.2227, and 0.1212, respectively. The study provides a new method for restoring occluded chicken areas that can hamper the success of vision-based machine predictions.

## 1. Introduction

The computer or machine vision-based technology (MVT) has been suggested and tested to monitor livestock and poultry behaviors [1,2,3,4], health [5,6], and flock activity [7,8,9]. At present, the techniques used to obtain information about poultry include 3D vision technology [10,11], infrared thermal imaging technology [12,13], and image processing technology [14,15,16]. 3D vision technology can effectively obtain the target area through the three-dimensional information in the scene. Aydin [17] used 3D vision technology to detect broilers and assessed the lameness of broiler chickens. 3D vision is more time-consuming than 2D vision because of the larger amount of data in 3D. Infrared thermal imaging technology uses temperature information to remove interferences and obtain poultry target areas. Zaninelli et al. [18] built an animal monitoring system based on infrared imaging technology and pattern recognition to detect a hen in a closed room of a housing system. Thermal imaging of poultry surface temperature is not consistent so that the low-temperature area tends to be lost. Image processing technology distinguishes target and non-target areas through image information characteristics. We used image processing technology to detect the area of the broiler chicken in the video scene and further analyzed the distribution of the broiler chicken [19]. Although the target area could be detected through image processing technology, it was dependent on the information in the scene. When the scene changes, the detection method may not be effective. In poultry houses, the complex production systems, such as feeding and drinking equipment (e.g., water pipe, feeder, and hanging chains), is a critical challenge for collecting top view animal images because animals or poultry are occluded in the images, which leads to the high uncertainty in analyzing animal information (e.g., behaviors and body features).

The poultry body is similar to an ellipse, so many researchers used ellipse fitting to obtain poultry information. Lao et al. [20] used contour ellipse fitting to obtain ten behavioral parameters. Further, the Naive Bayes Classification method has been used to classify and distinguish six behaviors of preening, shaking, resting, wing flapping, exploration, and wing lifting. Amraei et al. [21] performed ellipse fitting on the body of the chicken to obtain relevant parameters and conducted weight estimation through artificial neural networks. Poursaberi et al. [22] extracted the boundary of the bird and the parameters of the best-fitted ellipse to categorize turning, wing flapping, lying, and standing behaviors. In addition, the research on detecting elliptical targets has also achieved some results. Liu et al. [23] proposed a fast and effective ellipse detection method, which performed better detection results. Dong et al. [24] combined the advantages of arc extraction and arc grouping to propose an ellipse detection method. Therefore, the ellipse fitting method is one of the suitable methods for broiler chicken target detection.

The aforementioned methods can be modified to remove image interferences with ellipse fitting and obtain relevant chicken movement information. The objectives of this study were (1) develop an imaging processing strategy for removing equipment and restore occluded chicken areas; (2) test the effect of the optimized method to remove equipment areas; (3) evaluate the efficiency of different image restoration methods used in this study for two primary occlusion scenarios.

## 2. Materials and Methods

### 2.1. Experimental Setup and Data Collection

This study was conducted in two identical experimental broiler facilities on the Poultry Research Farm at the University of Georgia, Athens, USA. Unless otherwise stated, the experimental setup and data have been previously published in [19]. Briefly, six identical pens (measuring 1.84 L × 1.16 W m, 20 Cobb 500 broiler chickens/pen, the density was about 0.11 m^2^ floor per bird) were monitored separately with a high definition (HD) camera (PRO-1080MSFB, Swann Communications, Santa Fe Springs, CA, USA) mounted on the ceiling (2.5 m above floor) to capture video (15 frame/s with the resolution of 1440 × 1080 pixels). Video/image acquisition time was from 13 February 2020, to 18 March 2020. Collected videos were further analyzed and processed by MATLAB-R2019b (The MathWorks, Inc., Natick, MA USA).

### 2.2. Method for Target Detection

From our observation, the equipment interference in images of chickens became less with an increase in the birds’ size and age. Therefore, the first four weeks of chicken images were selected as research samples in this study. The method for target detection has been developed and published; see our other paper [19].

**Figure 1:**
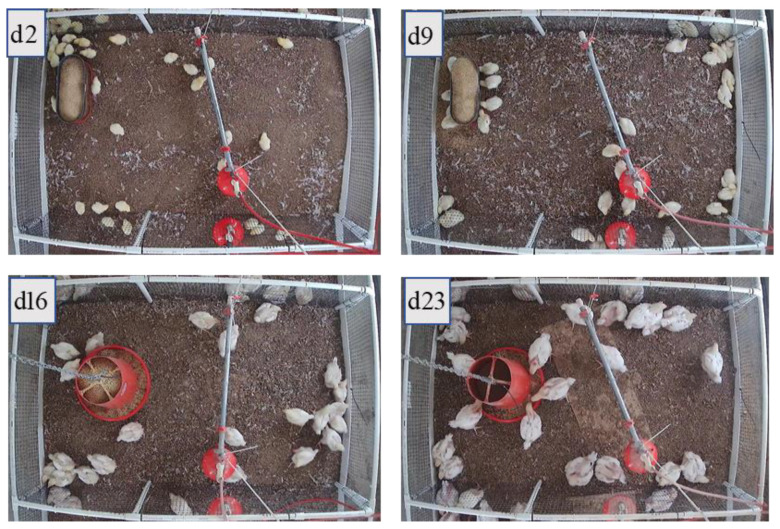
Examples of experimental data collection.

Figure 1 shows the images collected on d2, d9, d16, and d23. The hanging feeder was installed when birds were two weeks old and were tall enough to use it. Thus, images of d2 and d9 have a floor feeder, and d16 and d23 have a hanging feeder.

**Figure 2:**
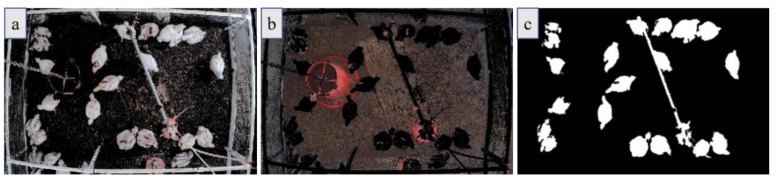
Examples of target detection results: (**a**) generation of binary image; (**b**) binary classification; and (**c**) the binary image obtained based on (**a**).

Figure 2 is an image collected on d23 as an example to show the target detection method. It can be seen from Figure 2c that the nipple drinker caused interference in birds’ detection. Therefore, it is necessary to remove this interference to improve the quality of the chicken’s images.

### 2.3. The Equipment Area Removal

According to pre-processing of images, we identified that image occlusion was caused by the presence of three pieces of equipment: (1) the water pipe; (2) the water pressure regulator (red circle area at the end of water pipe); and (3) the feeder. Therefore, the current study focused on the restoration of images occluded by the presence of three pieces of equipment.

(1) Water pipe interference removal.

**Figure 3:**
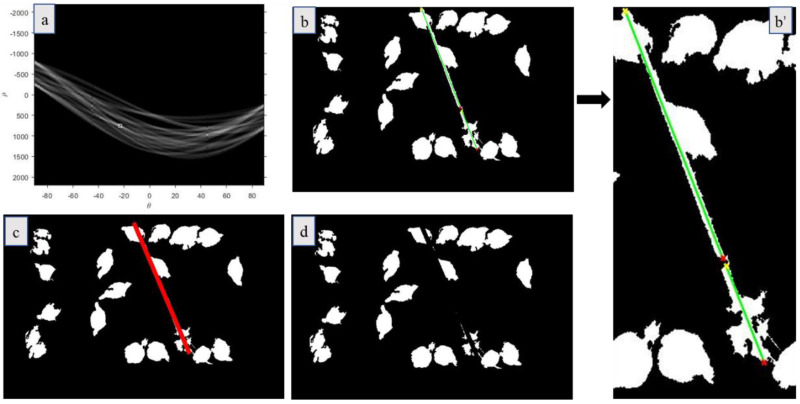
The process for pipe area detection and removal. The white box in (**a**) is the maximum peak point; In (**b**), it shows all the lines (green) that have passed the peak point with the yellow × indicating the starting point of the lines and the red × indicating the ending point of the lines. The drinking zone was zoomed-in (**b’**); In (**c**), the red area is the pipe area; and (**d**) is the result of pipe area removal.

The Hough transform can effectively detect the straight line in an image [25], so the method was used and modified to detect the pipe area in broiler houses. The Hough transform was performed on the image in Figure 2c to retain only the maximum peak (Figure 3a). Figure 3b,b’ show lines (green) that have passed the peak point with the ‘yellow ×’ indicating the starting point of the lines and the ‘red ×’ indicating the ending point of the lines. The first starting point in the ‘yellow ×’ was selected as the starting point of the pipe, and the last ending point in the ‘red ×’ was the ending point of the pipe. The connection line was approximately the centerline of the pipeline. Therefore, it was considered that the area obtained by the left and right extensions of 5 pixels based on the line was the pipe area, as shown in the red area in Figure 3c. Figure 3d shows the images with the pipe blocking area removed.

(2) Water pressure regulator interference removing.

**Figure 4:**
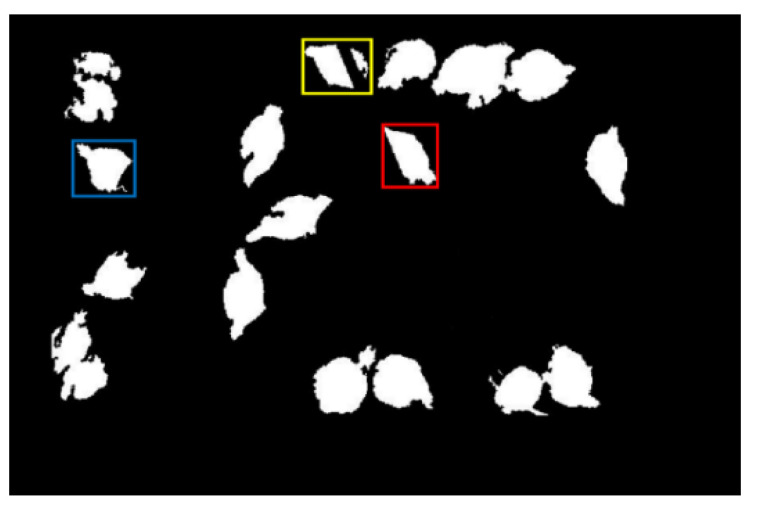
The result of equipment area removal. The area loss of the broiler chicken in the blue, yellow, and red boxes were caused by the feeder, water pipe, and water pressure regular, respectively.

The color of the water pressure regulator (i.e., circular area at the end of the water pipe in Figure 1) was red, which was quite different from the color of the broiler chickens. Therefore, the circular area of the water pressure regulator was removed by color information to obtain chicken profiles in Figure 4. It can be seen from Figure 4 that part of the broiler chicken’s missing area was caused by the occlusion of the water pipe, water pressure regulator, and feeder. For instance, the broiler chicken was divided into two areas in the yellow box in Figure 4.

### 2.4. Equipment Occlusion Detection and Restoration

**Figure 5:**
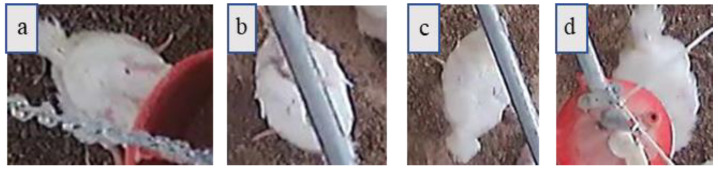
Examples of common equipment occlusions: (**a**) feeder occlusion; (**b**) water pipe occlusion–two areas (TA); (**c**) water pipe occlusion–one area (OA); (**d**) water pressure regulator occlusion.

Figure 5 shows the three equipment that occluded broiler chicken images, i.e., feeder (Figure 5a), water pipe (Figure 5b,c), and water pressure regulator (Figure 5d). There are two occlusion scenarios: (i) the body of chicken was divided into two areas (TA) (see Figure 5b) and (ii) the body of chicken was partly occluded, so the body has only one area (OA) (see Figure 5a,c,d). In this study, our method was modified to restore occlusion areas for both TA and OA scenarios.

(1) Image restoration for TA occlusion.

**Figure 6:**
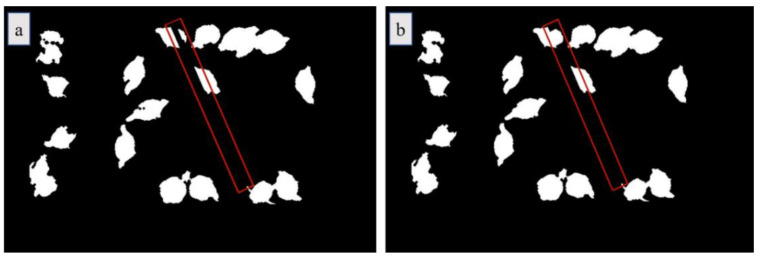
Occlusion area restoration for TA: (**a**) removed pipe and its surrounding area; (**b**) restored pipe area and chickens’ images.

To restore images of broiler chickens in the drinking zone, we removed the image background by keeping the water pipe and its surrounding area (i.e., red rectangular box area in Figure 6a). We performed a Linear Morphological Closure Operation (Linear Restoration Method) on the red box along the direction of the vertical water pipe, as shown in Figure 6b.

(2) Image restoration for OA occlusion.

Since the body shape of a broiler chicken is elliptical, the ellipse fitting method [26,27] was used to restore the occluded part of the broiler chicken. The ellipse fitting can be expressed using Equation (1).

(1) $$a\times x^{2}{\;+\;b\times y}^{2}\;+\;c\times x+\;d\times y\;+\;e\times x\times y\;+\;f\;=\;0$$

*a* ≠ 0, so Equation (2) can be changed to:

(2) $$x^{2}\;+\;\frac{b}{a}{\;\times \;y}^{2}\;+\frac{c}{\;a}\times \;x+\frac{d}{a}\times \;y\;+\;\frac{e}{a}\times \;x\;\times \;y\;+\frac{f}{a}=0$$

where *x*, *y* are variables, *x* is the abscissa of images, *y* is the ordinate of images. *a*, *b*, *c*, *d*, *e*, *f* are constants.

**Figure 7:**
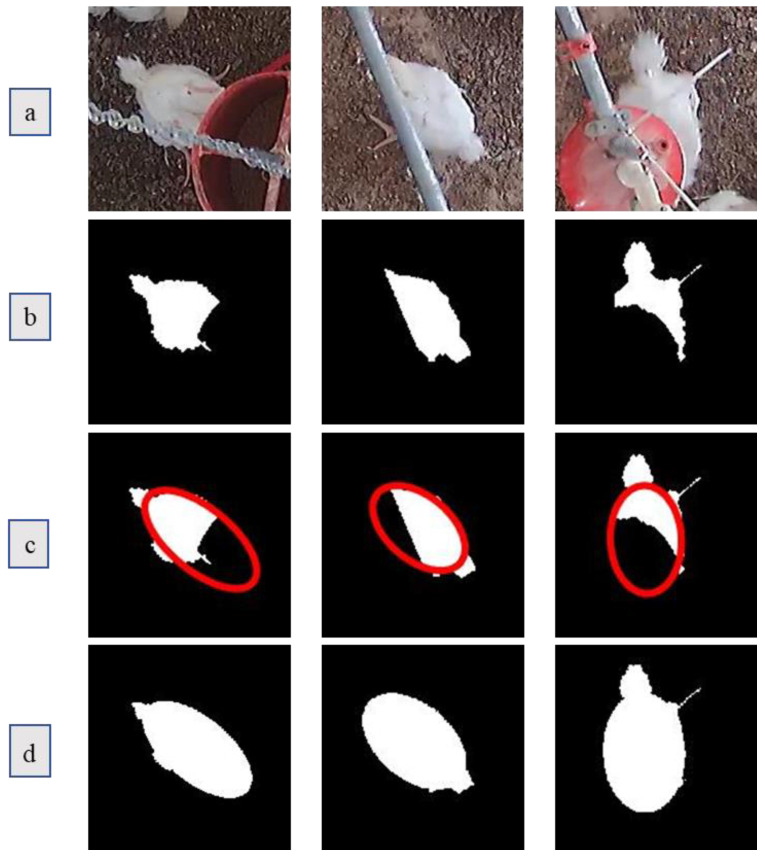
Example of the occlusion area restoration for OA: (**a**) is the original occlusion image; (**b**) is the corresponding detected binary image; (**c**) is the corresponding fitted ellipse (the red ellipse); and (**d**) is the restored result.

Five coordinates are needed to determine the ellipse. In this paper, the ellipse was calculated and obtained from five points selected from the boundary of the unobstructed body of the broiler chickens. Figure 7 shows the example how occlusion area under OA situation was restored. 

### 2.5. Evaluation Criteria and Statistical Analysis

Three evaluation indices were used to evaluate the image restoration methods: the overlap rate (OR), false-positive rate (FPR), and false-negative rate (FNR) [28].

The OR is the percentage of the actual target region affected by the overlapping of the actual target region and the restored target region. The higher the OR, the larger the overlap region and the better the restoration effect. The OR was calculated with Equation (3):

(3) $$\mathrm{OR}=\left(N_{1}\cap^{\;}N_{2}\right)/N_{1}\times 100\%$$

where *N*_1_ is the real region indicated by artificial marking and *N*_2_ is the region indicated by the restoration method.

The FPR is the percentage of the background region misjudged as the target region. The lower value, the better the restoration effect. The FPR was calculated with Equation (4):

(4) $$\mathrm{FPR}=\left[N_{2}-\left(N_{1}\cap^{\;}N_{2}\right)\right]/N_{1}\times 100\%$$

The FNR is the percentage of the target region misjudged as background. A lower value indicates a better effect of the restoration. The FNR was calculated with Equation (5):

(5) $$\mathrm{FNR}=\left[N_{1}-\left(N_{1}\cap^{\;}N_{2}\right)\right]/N_{1}\times 100\%$$

A one-way ANOVA (MATLAB-R2019b) was used to test if there were significant differences in OR, FPR, or FNR under different scenarios (e.g., one area and two areas) of occlusions. The effect was significant when the *p*-value was less than 0.05.

## 3. Results and Discussion

### 3.1. Restoration Efficiency for Occluded Area

**Figure 8:**
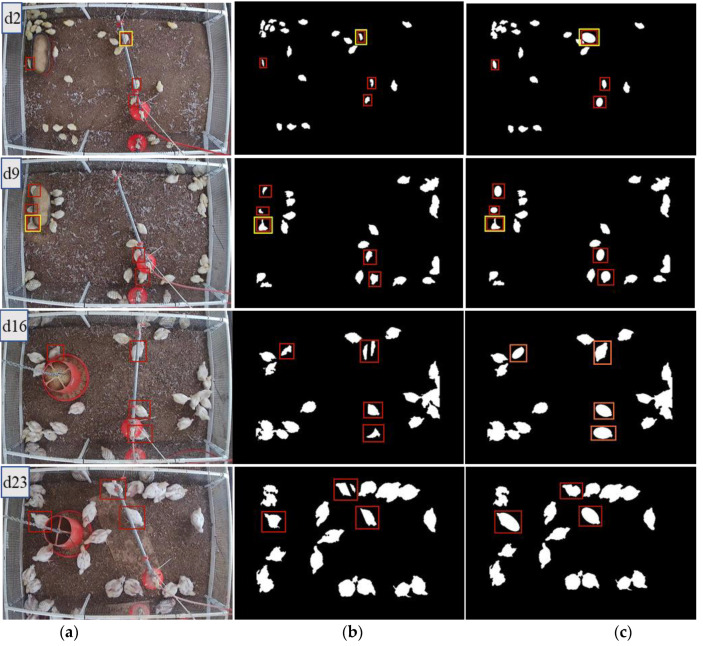
Restoration result of the occluded area. (**a**) original image; (**b**) the binary image of the broiler occluded area before restoration; (**c**) the binary image of the broiler occluded area after restoration. Red boxes are the broiler chickens occluded by the equipment. The yellow boxes are special cases, such as crowding.

About 100 images collected on d2, d9, d16, and d23 were selected for the new method evaluation. The restoration effect on occluded area of the chicken is shown in Figure 8, where red boxes are the broiler chickens occluded by the equipment. Basically, all images show occlusions, more or less.

It can be seen from Figure 8 that the broiler chicken area occluded by the equipment can be fixed. However, when the occlusion is extensive, such as the yellow box highlighted area (collected on d2) in Figure 8, the ellipse was overfitted. Therefore, we ascertained that it was not suitable to use ellipse fitting when chickens are crowded together (2 or more chickens in a group) and occluded by equipment (e.g., the yellow box of d9 in Figure 8 has two broiler chickens blocked by the feeder). In this study, we used ellipse fitting only when a single broiler chicken area was occluded.

To analyze the difference between the occluded area of chickens before and after image/occlusion restoration, the ratio of chicken area images before and after the restoration was quantified. Since the chicken posture was not uniform, the area of an individual chicken was determined by taking an average of the area of complete images of broiler chickens. From the d2, d9, d16, and d23 experimental images, about 150 broiler chicken area samples were obtained for TA and OA, respectively.

It can be seen from Table 1 that the linear restoration method can restore the occluded area well for the TA scenario, and there was a slight but not significant difference in the area compensation effect on different days/bird ages (*p* = 0.056). In this case, the occluded area of the pipe was relatively regular, so the restoration effect was superior. In the case of OA, there was no significant difference in the area compensation effect (*p* = 0.333) on different days/bird ages. We observed that the elliptical fitting restoration method can restore the occlusion area when the occlusion was not extensive better (e.g., when less than <50% of the broiler chicken area was occluded/blocked) (Figure 8; Table 1). When occlusion was extensive, ellipse underfitting or overfitting occurred (i.e., the restoration area was either too small or too large and likely contributed to the lack of significance in OA).

### 3.2. Performance of the Restoration Method

**Figure 9:**
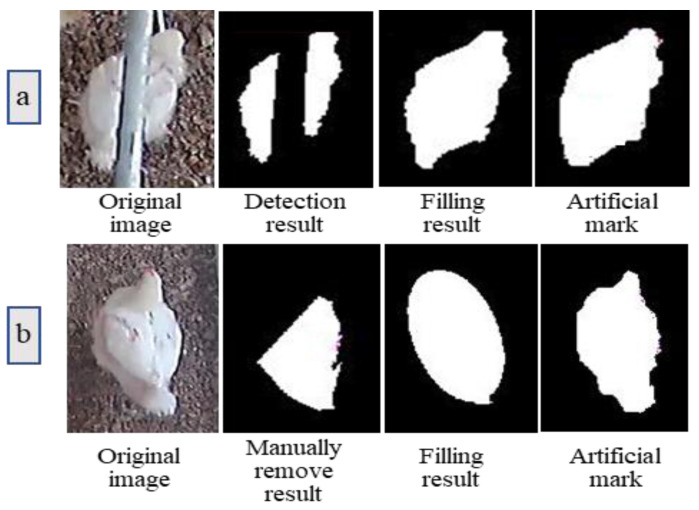
Comparison between restored chicken image area and an intact chicken image area (without occlusion): (**a**) process of image restoration for the TA situation; and (**b**) an intact chicken image without occlusion but artificially edited as the OA situation by removing a part for method evaluation.

When the occluded area of the broiler chicken could not be determined, predicting the actual area of the broiler chicken was not possible either. In the case of TA, the shape of the occluded area was regular (i.e., elliptical shape), so we could approximate the overall area of the broiler chicken as the actual area to evaluate the linear restoration method. Figure 9a shows the image with a bird blocked by water pipe (TA occlusion) and then reconstructed with the method developed in this study (i.e., ellipse fitting restoration). 

In the case of OA, the occluded area of the broiler chicken was irregular, which made it difficult to obtain the actual area of the broiler chicken. Therefore, to determine the complete broiler chicken area, we artificially removed some parts of the area to simulate occlusion and performed ellipse fitting restoration on the removed area to evaluate the restoration efficiency (Figure 9b). We selected 80 suitable target images from d2, d9, d16, and d23 broiler chicken images to determine the average values of OR, FPR, and FNR (Table 2).

It can be seen from Table 2 that for TA, the average values of OR, FPR, and FNR were 0.8150, 0.0032, and 0.1850, respectively. The smaller OR value and the larger FNR value of d2 and d9 were significantly different from other days (*p* < 0.05) because the broiler chickens were small, and the occluded area was relatively large, resulting in a large area of the broiler being lost. For the OR value and the FNR values of d16 and d23, there were no differences (*p* = 0.297). From Table 2, it can be concluded that as the broiler grows, the difference in linear restoration results decreases.

For OA, the average values of OR, FPR, and FNR at different ages were 0.8788, 0.2227, and 0.1212, respectively. Since the broiler chicken area was not elliptical, the ellipse fitting restores would classify part of the background area as the target area resulting in a larger FPR value (0.2227). The larger OR value (0.9106) and the smaller FNR value (0.0894) for d2 images was because the broiler chickens were small. Picking different points on the body boundary resulted in different ellipses, which affected the restoration result, thereby leading to a lack of significance in OR in images on four different days (*p* = 0.111), in FPR in images of d2, d9, and d16 (*p* = 0.082), and FNR in images of four different days (*p* = 0.111).

In the current study, the selection of 5-points needs further improvement to optimize the ellipse fitting. In addition, monitoring individual poultry behaviors (e.g., feeding, drinking, lying, standing, walking, etc.) needs to be studied separately in occlusion restoration because behavior postures are different from each other. We developed a method based on the shape feature of the broiler chicken (ellipse) to restore the occluded area of the broiler chickens. In addition, some other machine learning or deep learning algorithms, such as support vector machines, have been reported with a similar function in image processing. Comparing ellipse fitting to other machine learning or deep learning algorithms/models is required to develop or optimize the method for occlusion restoration and other automatic methods for poultry behaviors or health monitoring.

## 4. Summary and Conclusions

In this study, a machine vision-based method was optimized to restore broiler chickens’ images occluded by an equipment. According to the pre-processing of images, the general occlusion was identified as two area occlusion (TA) and one area (OA) occlusion. Three evaluation indices include the overlap rate (OR), false-positive rate (FPR), and false-negative rate (FNR), were used to evaluate the restoration method.

For TA occlusion, the average values of OR, FPR, and FNR were 0.8150, 0.0032, and 0.1850, respectively. The linear restoration effect was better than elliptical fitting, which was less affected by the growth of the broiler chicken because the occluded area was regular/normal in the case of TA. For OA occlusion, the average values of OR, FPR, and FNR were 0.8788, 0.2227, and 0.1212, respectively. The method we optimized/developed was not applicable for some special situations, such as crowding.

In the future study, the occluded area of crowded broiler chickens will be segmented first and then restored. In addition, monitoring individual poultry behaviors (e.g., feeding, drinking, lying, standing, walking, etc.) needs to be studied separately in occlusion removal because behavior postures are different from each other.

## Figures and Tables

**Table 1:** The ratio of a broiler chicken area occluded by the equipment before and after restoration compared to an intact broiler chicken area (150 samples each).

Occlusion Type	d2		d9		d16		d23	
	BF_area_/IN_aera_	AF_area_/IN_aera_	BF_area_/IN_aera_	AF_area_/IN_aera_	BF_area_/IN_aera_	AF_area_/IN_aera_	BF_area_/IN_aera_	AF_area_/IN_aera_
TA	0.4357	0.9706	0.4971	0.9687	0.6299	0.9637	0.6747	0.9512
OA	0.4773	1.2008	0.4518	0.9445	0.5077	1.009	0.6962	1.1017

**Note**: BF_area_ is the average area of broiler chickens before restoration; AF_area_ is the average area of broiler chickens after restoration; IN_aera_ is the area of the intact broiler chicken area (not occluded by an equipment). TA—two areas; OA—one area.

**Table 2:** Comparison of average values on different days.

Evaluation Indices	Occlusion Type	d2	d9	d16	d23	Average
OR	TA	0.7265 ^a^	0.8240 ^b^	0.8593 ^c^	0.8502 ^c^	0.8150
	OA	0.9106 ^a^	0.8480 ^a^	0.8673 ^a^	0.8834 ^a^	0.8788
FPR	TA	0.0022 ^a^	0.0076 ^b^	0.0031 ^c^	0.0002 ^d^	0.0032
	OA	0.2963 ^a^	0.2216 ^ab^	0.2064 ^ab^	0.1665 ^b^	0.2227
FNR	TA	0.2735 ^a^	0.1760 ^b^	0.1407 ^c^	0.1498 ^c^	0.1850
	OA	0.0894 ^a^	0.1520 ^a^	0.1327 ^a^	0.1166 ^a^	0.1212

**Note**: In the same row, different letters of a, b, c and d represent significant differences among the means (*p* ≤ 0.05); Evaluation indices include the overlap rate (OR), false-positive rate (FPR), and false-negative rate (FNR).

## Data Availability

Not applicable.
